# The effects of acarbose therapy on reductions of myocardial infarction and all-cause death in T2DM during 10-year multifactorial interventions (The Beijing Community Diabetes Study 24)

**DOI:** 10.1038/s41598-021-84015-0

**Published:** 2021-03-01

**Authors:** Xue-Lian Zhang, Shen-Yuan Yuan, Gang Wan, Ming-Xia Yuan, Guang-Ran Yang, Han-Jing Fu, Liang-Xiang Zhu, Jian-Dong Zhang, Yu-Ling Li, Da-yong Gao, Xue-Li Cui, Zi-ming Wang, Rong-Rong Xie, Ying-jun Chen

**Affiliations:** 1 Department of Endocrinology, Beijing Tongren Hospital, Capital Medical University, 1 Dong Jiao Min Xiang, Beijing, 100730 China; 2 Medical Records and Statistics Department, Beijing Ditan Hospital, Capital Medical University, Beijing, China; 3 Department of Endocrinology, Beijing Friendship Hospital, Capital Medical University, Beijing, China; 4 Jinsong Community Health Service Center, Beijing, China; 5 Xinjiekou Community Health Service Center, Beijing, China; 6 Aerospace Central Hospital, Beijing, China; 7 Sanlitun Community Health Service Center, Beijing, China; 8 Jiangtai Community Health Service Center, Beijing, China; 9 Majiapu Community Health Service Center, Beijing, China

**Keywords:** Diabetes complications, Interventional cardiology, Drug delivery

## Abstract

To investigate the potential benefits of acarbose therapy on cardiovascular events (CVD) in Type 2 diabetes (T2DM) in an urban community over 10-year follow-up. The study population of Beijing Community Diabetes Study (BCDS) were type 2 diabetes (T2DM) living in 21 communities in Beijing. All patients received comprehensive intervention in accordance with the Chinese guidelines for the prevention and treatment of diabetes. Professors in endocrinology from top tier hospitals regularly visited the communities for consultations, which was a feature of this study. A total of 1797 T2DM in BCDS study had complete screening data, including blood glucose, blood pressure, lipid profiles and acarbose continuous therapy. After 10-year follow-up, the risks of CVD outcomes were assessed according to whether patients had received acarbose therapy or not. All patients were followed-up to assess the long-term effects of the multifactorial interventions. At baseline, compared with the acarbose therapy free in T2DM, there was no significant difference in achieving the joint target control in patients with acarbose therapy. From the beginning of 8th year follow-up, the joint target control rate in patients with acarbose therapy was significantly higher than that of acarbose therapy free. During the 10-year follow-up, a total of 446 endpoint events occurred, including all-cause death, cardiovascular events, cerebrovascular events. The incidences of myocardial infarction (from the 4th year of follow-up) and all-cause death (from the 2nd year of follow-up) in patients who received acarbose therapy were significantly lower than that of acarbose therapy free respectively. In Cox multivariate analyses, there were significant differences in incidences of myocardial infarction and all-cause death between afore two groups during the 10-year follow-up, and the adjusted HRs were 0.50 and 0.52, respectively. After multifactorial interventions, T2DM with acarbose therapy revealed significant reductions of myocardial infarction and all-cause death. The long-term effects of with acarbose therapy on improving joint target control might be one of the main reasons of myocardial infarction and all-cause death reduction.

**Trial Registration**: ChiCTR-TRC-13003978, ChiCTR-OOC-15006090.

## Introduction

In 2013, a Chinese nationwide survey by Wang et al. on diabetes epidemiology showed that the prevalence of diabetes in China reached 10.9%. Among them, only 32.2% of the patients received diabetes treatment, and the control rate of blood glucose was only 49.2%^1^. Since hyperglycemia can cause nephropathy, neuropathy, retinopathy and macrovascular diseases^2,3^, the poor control rate of blood glucose should be improved.

Collaborative Analysis of Diagnostic Criteria in Europe (DECODE) study showed that the correlation of 2-h postprandial blood glucose with coronary heart disease and total mortality was closer than that of fasting blood glucose^4^.

Previous study had shown that there was a 49% risk reduction in cardiovascular events in subjects with impaired glucose tolerance (IGT) after acarbose treatment^5^. In a meta-analysis, acabose users in Type 2 diabetes (T2DM) were associated with a 35% reduction in cardiovascular events^6^. The mechanisms might be putatively attributed to the postprandial hyperglycemia demission, which was benefit for a lowering of reaction of oxidative stress^7^. However, the above-mentioned studies were investigated in patients with IGT^5^, short-term studies^8^, or meta-analysis^9^.

The UK prospective diabetes study (UKPDS), which is a first clinical trial on intensive control of blood glucose in newly diagnosed T2DM for 20 years. After prolonged tracking until 30 years, the results showed that intensive control of blood glucose could significant reduce myocardial infarction. Although there as in small sample of T2DM combined with microalbuminuria, after intensified multifactorial intervention, the results of Steno-2 study showed the reduction in the risk of cardiovascular disease and prolonged life over 21.2 years of follow-up. So far, no large-scale, prospective intervention clinical studies have been found to affect myocardial infarction.

As we all known, the α-glucosidase inhibitors were commonly used as a kind of antidiabetic agents, either for monotherapy in mild diabetes, or for more advanced diabetes in combination with other antidiabetic agents, especially in China^10^. But there were no population-based long-term investigations about the potential cardiovascular effect of acarbose treatment in patients with T2DM in China.

To address this issue, we designed the first 10-year Community Follow-up Study in China. Patients who were diagnosed with T2DM were enrolled from 21 communities of Beijing.

## Methods

### Study population and intervention methods

Details of the BCDS study have been published previously^11^. For the present study (BCDS 23), a total of 21 communities of residents were recruited between August 2008 and July 2009. They were aged between 20 and 80 years, and all had been diagnosed with T2DM. The study was conducted according to the declaration of Helsinki. The study was approved by the Medical Ethics Committee of Beijing Tongren Hospital and all participants provided written informed consent.

The patients enrolled in BCDS were monitored during the 10-year multi-factorial intervention^12^. Goals of treatment in BCDS are in accordance with 2007 and 2010 China guideline for T2DM. The joint target control was defined as HbA1c < 7.0%, systolic blood pressure (SBP) < 130 mmHg, diastolic blood pressure (DBP) < 80 mmHg, and low-density lipoprotein cholesterol (LDL-C) < 2.6 mmol/L (with coronary heart disease < 1.8 mmol/L).

Approaches to prevention of diabetic complications in BCDS include the following: patients were seen at clinic visits every 2 months; HbA1c every 3–6 months; Annual micro-albumin checks^11^; yearly dilated eye examinations; measurements of the lipid profile and ECGs were performed 6-monthly or yearly.

All patients were diagnosed with T2DM according to the World Health Organization diagnostic criteria, which combined with or without CVD. The diagnosis of CVD was made by top tier hospitals, including cerebrovascular disease, coronary heart disease, myocardial infarction, angina pectoris, or other CVD clinical indications. Cerebrovascular events in this study included stroke (acute cerebral infarction with limb disorders, acute cerebral hemorrhage) and transient ischemic attack (TIA). The diagnostic basis for hypertension included self-reported use of antihypertensive drugs and/or systolic blood pressure ≥ 140 mmHg and/or diastolic blood pressure ≥ 90 mmHg.

**Figure 1:**
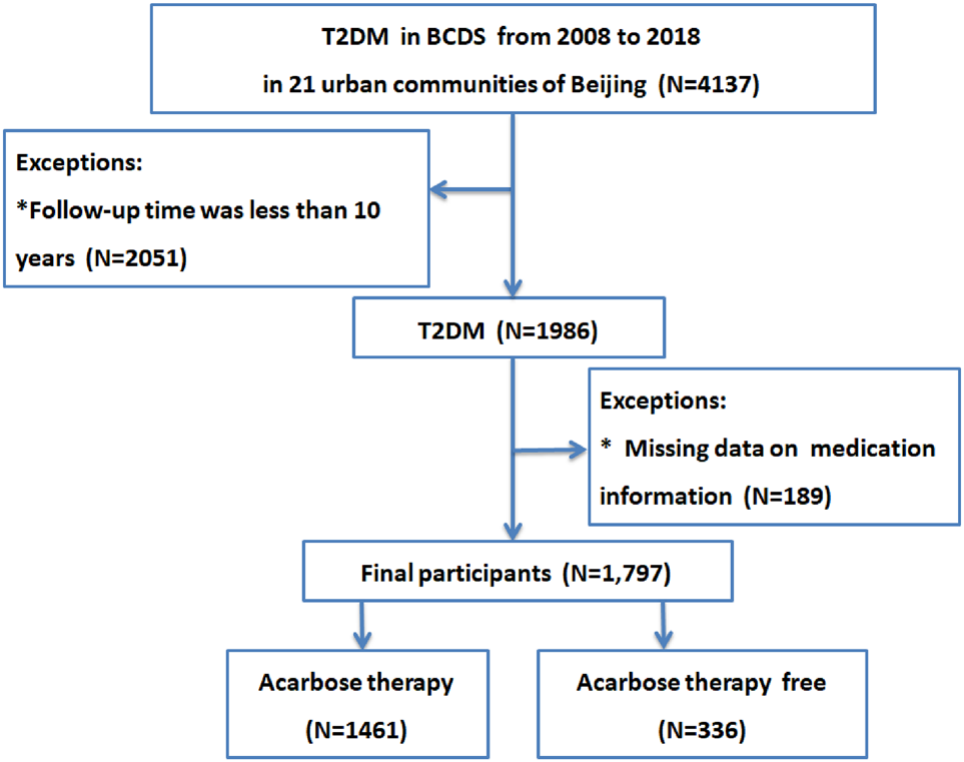
Flow chart showing the number of study participants.

A total of 1796 adults with T2DM were enrolled for analysis who have complete screening data, including blood glucose, blood pressure, lipid profiles and acarbose therapy. Among them, 1461 patients received acarbose therapy while 336 patients did not receive acarbose therapy (Fig. 1). All patients were followed up regularly to monitor the relevant indexes and recorded actual CVD events. Subsequently, the effects of the acarbose therapy in reducing the cardiovascular events were evaluated by comparing joint target control rates with that of baseline rates.

All-cause endpoints were adjudicated by an independent committee, which were in charge of assignments on validation of data and events based on the endpoint events criteria. For the present study, CVD outcomes were defined as the first nonfatal or fatal cardiac and cerebral vascular events, including sudden cardiac death, myocardial infarction, and nonfatal stroke. The diagnosis of new myocardial infarction, stroke, or angina pectoris was based on the following evidences, including diagnostic certificate issued by specialist from top tier hospital. Myocardial infarction was defined according to symptoms, electrocardiogram, and biomarkers of necrosis. Angina was defined according to the presence of symptoms and objective demonstration of ischemia on ECG or presence of coronary stenosis. And coronary revascularization, including percutaneous invasive revascularization and surgery. All cerebrovascular events were confirmed by cranial computed tomography or brain magnetic resonance imaging examination or cerebrovascular imaging.

Patients excluded from analysis in the present study included those with type 1 diabetes, urinary infections, hematuria, severe disabilities, hepatic failure, renal failure, schizophrenia, goiter or fever, sleep apnea syndrome.

### Study procedures

Baseline information on sociodemographic variables were collected^11^, included age, gender, smoking habits, history of diabetes, and parental history of diabetes. Data obtained from clinical evaluations included height, body weight, body mass index (BMI), and waist circumference (WC) and hip circumference (HC). The neck circumference (NC) at the upper margin of the laryngeal prominence was also measured with the patients’ heads erect and eyes facing forward. Systolic blood pressure (SBP) and diastolic blood pressure (DBP) values in all participants were also obtained.

### Laboratory data

Laboratory measurements (using established methods) were checked in all T2DM, included fasting plasma glucose (FPG), serum lipid concentrations (total cholesterol, alanine aminotransferase (ALT), blood urea nitrogen, serum creatinine concentrations, low- and high-density lipoprotein [LDL and HDL]-cholesterols, and triglycerides). HbA1c (%) was measured using a Bio-Rad Variant hemoglobin analyzer by a central endocrinology laboratory in Beijing Tongren Hospital. Nonstereoscopic photographs of the central fundus were taken for all eyes (Camera CR-DGi; Canon Inc, Tokyo, Japan)^11^.

All patients underwent a resting 12-lead ECG. Angina pectoris and/or > 0.1 mV ST segment deviation during a treadmill maximal exercise stress test were recorded. Ischemic heart disease was diagnosed based on the results of coronary angiography.

On December 31, 2018, participants were informed of the final results and asked to continue with normal medical care every year.

### Statistical analyses

The BCDS database was set up using network database. Statistical analysis was performed using SAS software (version 9.2, SAS Institute Inc., Cary, USA). The results were expressed as means (± SD) or median (Q1, Q3). Based on whether the patients were acarbose users or not, age, gender, clinical comorbidities, and concomitant use of other diabetes medications were compared between two groups using χ^2^ 2-tests for categorical variables and Student’s t-tests for continuous variables.

We used the Cox proportional hazards analysis to estimate hazards ratios (HRs) of cardiovascular risk factors, with 95% confidence interval (CI) for the effects of acarbose on CVD risk. We included the clinically important factor such as age, gender, blood pressure, NC, and lipid profiles, etc. Kaplan–Meier analysis was used to assess the cumulative percent of CVD events between acarbose users and non-users by follow-up time, and then log-rank test was used to assess the difference between two groups. All tests were two-sided, and the level of significance was established as P-values < 0.05.

### Ethics approval and consent to participate

The study was conducted according to the declaration of Helsinki. The Medical Ethics Committee of Beijing Tongren Hospital approved the study protocol, and all participants provided their written informed consent.

## Results

### Patients’ clinical characteristics

The baseline clinical characteristics are shown in Table 1. 1796 patients with T2DM were included in the analysis, which were divided into two groups based on whether receive acarbose therapy (1461 cases, 81.3%) or not (336 cases, 19.7%). Among them, 1461 cases were treated with acarbose, either as monotherapy or in combination with other antidiabetic regimens.

**Table 1:** Comparison of demographic and clinical characteristics between acarbose therapy free and acarbose therapy at baseline (2008) in the Beijing Communities Diabetes Study (Report 24).

Characteristic	Total	Acarbose therapy free	Acarbose therapy	Statistical quantity	P
	(n = 1797)	(n = 336)	(n = 1461)		
Age (years)	64.36 ± 9.71	64.15 ± 10.48	64.41 ± 9.52	− 0.42 (t)	0.674
**Gender**					
Male, n (%)	684 (38.06)	140 (41.67)	544 (37.23)	2.28 (χ^2^)	0.131
Female, n (%)	1113 (61.94)	196 (58.33)	917 (62.77)		
Duration of diabetes (years)	7.01 ± 6.69	5.0 (1.0, 11.3)	5.3 (1.5, 10.8)	0.72 (Z)	0.470
**Educational attainment**					
Low, n (%)	296 (16.63)	58 (17.52)	238 (16.43)	8.66 (χ^2^)	0.013
Medium, n (%)	1153 (64.78)	194 (58.61)	959 (66.18)		
High, n (%)	331 (18.60)	79 (23.87)	252 (17.39)		
**Economic level (monthly income)**					
~ 1000¥, n (%)	116 (6.89)	20 (6.51)	96 (6.97)	3.78 (χ^2^)	0.286
1000–2000¥, n (%)	877 (52.08)	158 (51.47)	719 (52.21)		
2000–4000¥, n (%)	593 (35.21)	104 (33.88)	489 (35.51)		
4000¥~, n (%)	98 (5.82)	25 (8.14)	73 (5.30)		
Height (cm)	162.34 ± 7.61	162.84 ± 7.83	162.22 ± 7.55	1.35 (t)	0.178
Weight (kg)	66.62 ± 10.94	67.49 ± 10.47	66.43 ± 11.04	1.61 (t)	0.109
Body mass index (kg/m^2^)	25.23 ± 3.43	25.41 ± 3.25	25.19 ± 3.47	1.08 (t)	0.280
Waist circumference (cm)	88.19 ± 9.13	88.89 ± 8.69	88.02 ± 9.22	1.57 (t)	0.117
Hip circumference (cm)	99.21 ± 8.93	99.60 ± 8.84	99.12 ± 8.96	0.88 (t)	0.377
Waist–hip ratio	0.89 ± 0.06	0.89 ± 0.06	0.89 ± 0.06	1.28 (t)	0.202
Neck circumference (cm)	36.19 ± 3.83	36.60 ± 3.90	36.10 ± 3.81	2.18 (t)	0.029
Systolic blood pressure (mmHg)	129.36 ± 13.26	129.40 ± 12.94	129.35 ± 13.33	0.06 (t)	0.950
Diastolic blood pressure (mmHg)	77.29 ± 8.26	77.81 ± 7.81	77.18 ± 8.36	1.27 (t)	0.203
**Smoker**					
No, n (%)	1558 (86.85)	290 (86.31)	1268 (86.97)	0.10 (χ^2^)	0.747
Yes, n (%)	236 (13.15)	46 (13.69)	190 (13.03)		
FPG (mmol/L)	7.24 ± 3.12	7.05 ± 3.37	7.29 ± 3.06	− 1.16 (t)	0.249
Hpg (mmol/L)	10.73 ± 4.24	10.77 ± 4.33	10.72 ± 4.22	0.19 (t)	0.852
HbA1c (%)	7.34 ± 1.47	7.27 ± 1.44	7.35 ± 1.48	− 0.91 (t)	0.365
Triglycerides (mmol/L)	1.68 ± 1.35	1.77 ± 1.71	1.65 ± 1.26	1.13 (t)	0.259
Total cholesterol (mmol/L)	4.72 ± 1.75	4.67 ± 1.84	4.73 ± 1.73	− 0.55 (t)	0.584
HDL-cholesterol (mmol/L)	1.21 ± 0.60	1.21 ± 0.81	1.21 ± 0.54	0.01 (t)	0.993
LDL-cholesterol (mmol/L)	2.76 ± 1.17	2.74 ± 1.20	2.77 ± 1.16	− 0.42 (t)	0.671
Alanine aminotransferase (U/L)	22.64 ± 14.08	21.69 ± 11.46	22.85 ± 14.60	− 1.43 (t)	0.154
Blood urea nitrogen (mmol/L)	5.8 (4.8, 7.2)	6.0 (5.0, 7.4)	5.7 (4.7, 7.2)	2.33 (Z)	0.020
Creatinine (mmol/L)	65.4 (53.5, 79.0)	66.0 (54.0, 81.6)	65.0 (53.5, 78.8)	1.19 (Z)	0.234
Urine acid (mmol/L)	286.7 (229.9, 347.2)	286.0 (228.0, 342.5)	286.7 (230.0, 349.0)	0.45 (Z)	0.650
**Antihypertensive therapy**					
No, n (%)	663 (36.89)	128 (38.10)	535 (36.62)	0.26 (χ^2^)	0.613
Yes, n (%)	1134 (63.11)	208 (61.90)	926 (63.38)		
**Hypoglycemic therapy**					
No, n (%)	141 (7.85)	42 (12.50)	99 (6.78)	12.38 (χ^2^)	< 0.001
Yes, n (%)	1656 (92.15)	294 (87.50)	1362 (93.22)		
**Metformin**					
No, n (%)	1300 (72.34)	243 (72.32)	1057 (72.35)	0.00 (χ^2^)	0.992
Yes, n (%)	497 (27.66)	93 (27.68)	404 (27.65)		
**Alpha-glucosidase inhibitor**					
No, n (%)	945 (52.59)	336 (100.0)	609 (41.68)	372.60 (χ^2^)	< 0.001
Yes, n (%)	852 (47.41)	0 (0.00)	852 (58.32)		
**Sulfonylurea drugs**					
No, n (%)	1041 (57.93)	182 (54.17)	859 (58.80)	2.40 (χ^2^)	0.121
Yes, n (%)	756 (42.07)	154 (45.83)	602 (41.20)		
**Insulin therapy**					
No, n (%)	1402 (78.02)	238 (70.83)	1164 (79.67)	12.44 (χ^2^)	< 0.001
Yes, n (%)	395 (21.98)	98 (29.17)	297 (20.33)		
**Hypolipidemic drug**					
No, n (%)	573 (31.89)	107 (31.85)	466 (31.90)	0.00 (χ^2^)	0.986
Yes, n (%)	1224 (68.11)	229 (68.15)	995 (68.10)		
**Statins**					
No, n (%)	1487 (82.75)	287 (85.42)	1200 (82.14)	2.06 (χ^2^)	0.151
Yes, n (%)	310 (17.25)	49 (14.58)	261 (17.86)		
**History of disease**					
Cardiac and cerebral vascular disease					
No, n (%)	1261 (70.17)	233 (69.35)	1028 (70.36)	0.14 (χ^2^)	0.713
Yes, n (%)	536 (29.83)	103 (30.65)	433 (29.64)		
Cerebral vascular disease					
No, n (%)	1565 (87.09)	297 (88.39)	1268 (86.79)	0.62 (χ^2^)	0.429
Yes, n (%)	232 (12.91)	39 (11.61)	193 (13.21)		
Coronary heart disease					
No, n (%)	1410 (78.46)	262 (77.98)	1148 (78.58)	0.06 (χ^2^)	0.809
Yes, n (%)	387 (21.54)	74 (22.02)	313 (21.42)		
Malignant tumor					
No, n (%)	1756 (97.72)	328 (97.62)	1428 (97.74)	0.02 (χ^2^)	0.892
Yes, n (%)	41 (2.28)	8 (2.38)	33 (2.26)		
Nephropathy					
No, n (%)	1738 (96.72)	325 (96.73)	1413 (96.71)	0.00 (χ^2^)	0.991
Yes, n (%)	59 (3.28)	11 (3.27)	48 (3.29)		
Retinopathy					
No, n (%)	1710 (95.16)	320 (95.24)	1390 (95.14)	0.01 (χ^2^)	0.940
Yes, n (%)	87 (4.84)	16 (4.76)	71 (4.86)		

N = number of individuals. Values are expressed as mean ± SD, median (Q1, Q3) or number (%). P statistical significance of the differences between the two groups.

*BMI* body mass index, *SBP* systolic blood pressure, *DBP* diastolic blood pressure, *TG* triglyceride, *TC* total cholesterol, *HDL-C* high density lipoprotein cholesterol, *LDL-C* low density lipoprotein cholesterol, *FPG* fasting plasma glucose, *Hpg* 2-h postprandial blood glucose, *HbA1c* hemoglobin A1c.

The clinical characteristics, such as age, gender, the frequency of smoking, comorbidities and combination treatment with other oral antidiabetic regimens and use of statins were all similar between the two groups (Table 1). There were no significant differences in weight and BMI between the two groups, while the neck circumference was significant higher in patients with acarbose therapy free than that of acarbose therapy. At baseline, there were no significant differences in blood glucose (including fasting blood glucose, postprandial blood glucose and HbA1c) between the two groups.

Results of micro-vascular complications in diabetes will be published in other articles. Educational attainment was categorized into three levels: low (illiteracy or elementary school), medium (middle school), and high (college or academic degree). According to educational attainment, compared with patients with acarbose therapy free, the acarbose users were well educated, and had more use of insulin therapy (P < 0.01) (Table 1).

During the follow-up, the fasting blood glucose and HbA1c levels were significant higher in patients with acarbose therapy than that of acarbose therapy free (P < 0.05). The postprandial blood glucose was similarly between the two groups. In addition to blood glucose control, compared with the acarbose therapy free, there were no significant differences in clinical control of blood pressure. Although the proportion of hypolipidemic drug used (including Statins) in the two groups was different at the end of the follow-up, dyslipidemia was similar between the two groups (Table 2). After multi-factorial invention for 10 years, many metabolic indicators between the two groups have been improved, including blood glucose, blood pressure, and LDL-cholesterols (Table 2).

**Table 2:** Comparison of clinical characteristics between acarbose therapy free and acarbose therapy at end of study follow up (2018) in the Beijing Communities Diabetes Study (Report 24).

	Acarbose therapy free	Acarbose therapy	Statistical quantity	P
Body mass index (kg/m^2^)	25.18 ± 3.16	24.98 ± 3.13	0.72 (t)	0.475
Waist circumference (cm)	88.26 ± 8.33	87.30 ± 8.49	1.29 (t)	0.196
Hip circumference (cm)	98.11 ± 7.96	97.40 ± 8.17	1.00 (t)	0.316
Waist–hip ratio	0.90 ± 0.05	0.90 ± 0.05	0.67 (t)	0.500
Neck circumference (cm)	36.67 ± 3.43	36.31 ± 3.93	1.19 (t)	0.236
Systolic blood pressure (mmHg)	125.04 ± 8.50	126.09 ± 8.34	− 1.43 (t)	0.152
Diastolic blood pressure (mmHg)	73.05 ± 5.79	73.12 ± 6.47	− 0.11 (t)	0.910
FPG (mmol/L)	7.31 ± 1.62	7.80 ± 4.56	− 2.52 (t)	0.012
Hpg (mmol/L)	9.13 ± 2.56	9.06 ± 2.09	0.28 (t)	0.776
HbA1c(%)	6.85 ± 1.05	7.08 ± 1.17	− 2.17 (t)	0.030
Triglycerides (mmol/L)	1.56 ± 0.80	1.57 ± 0.80	− 0.10 (t)	0.917
Total cholesterol (mmol/L)	4.46 ± 0.81	4.49 ± 0.98	− 0.48 (t)	0.629
HDL-cholesterol (mmol/L)	1.33 ± 0.65	1.28 ± 0.31	0.97 (t)	0.332
LDL-cholesterol (mmol/L)	2.38 ± 0.64	2.48 ± 0.75	− 1.62 (t)	0.108
Alanine aminotransferase (U/L)	20.58 ± 10.68	20.62 ± 10.96	− 0.04 (t)	0.967
Blood urea nitrogen (mmol/L)	5.6 (5.1, 6.5)	5.8 (5.1, 6.8)	1.03 (Z)	0.304
Creatinine (mmol/L)	72.0 (63.3, 81.5)	71.0 (61.0, 82.0)	1.05 (Z)	0.295
Urine acid (mmol/L)	311.0 (247.0, 356.0)	312.0 (259.0, 365.0)	1.14 (Z)	0.255
**Hypolipidemic drug**				
No, n (%)	65 (19.35)	175 (11.98)	12.81 (χ^2^)	0.000
Yes, n (%)	271(80.65)	1286 (88.02)		
**Statins**				
No, n (%)	191 (56.85)	529 (36.21)	48.45 (χ^2^)	< 0.001
Yes, n (%)	145 (43.15)	932 (63.79)		

N = number of individuals. Values are expressed as mean ± SD, median (Q1, Q3) or number (%). P statistical significance of the differences between the two groups.

*BMI* body mass index, *SBP* systolic blood pressure, *DBP* diastolic blood pressure, *TG* triglyceride, *TC* total cholesterol, *HDL-C* high density lipoprotein cholesterol, *LDL-C* low density lipoprotein cholesterol, *FPG* fasting plasma glucose, *Hpg* 2-h postprandial blood glucose, *HbA1c* hemoglobin A1c.

At the end of follow-up, compared with the baseline, the mean levels of 2-h postprandial blood glucose and HbA1c decreased in patients with acarbose therapy free and acarbose users (1.64 mmol/L vs. 1.66 mmol/L; 0.42% vs. 0.27%), while the fasting blood glucose levels increase in two groups. The average levels of SBP and DBP decreased in the two groups (4.36 mmHg vs. 3.26 mmHg, 4.76 mmHg vs. 4.06 mmHg) respectively. For some lipid variables, TC and LDL-C levels also reduced (0.21 mmol/Lvs.0.32 mmol/L; 0.36 mmol/L vs. 0.39 mmol/L) in acarbose therapy free and acarbose treatment. The improvement of body weight showed that BMI reduced in non-users of acabose and acarbose users (0.23 kg/m^2^ vs. 0.21 kg/m^2^) at the end of follow-up (Table 2).

### Effect of acarbose therapy on patients’ joint target control

**Figure 2:**
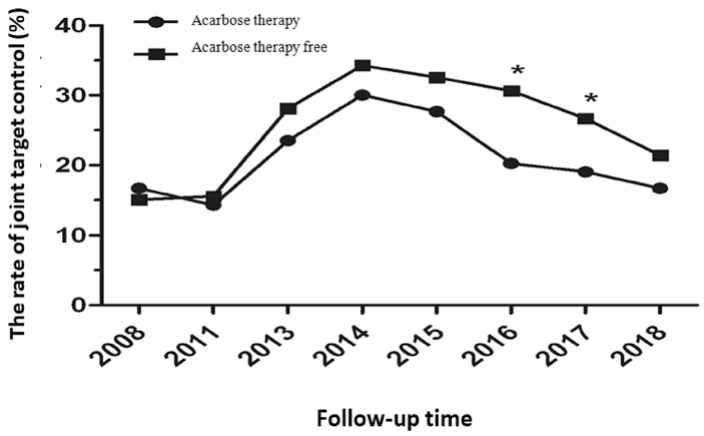
Changes of joint target control during 10-year follow-up between the two groups.

At baseline, compared with the acarbose therapy free in T2DM, there was no significant difference in achieving the joint target control in patients with acarbose therapy. From the beginning of the 8th year of follow-up, the patients with acarbose therapy were more likely to have higher joint target control ratios than that of acarbose therapy free (*P* < 0.01) (Table 3, Fig. 2).

**Table 3:** Comparison of ratios of patients who achieved the joint target control between the two groups.

Characteristic	Total	Acarbose therapy free	Acarbose therapy	Statistical quantity	P
	(n = 1797)	(n = 336)	(n = 1461)		
**Joint target control in 2008**					
Yes, n (%)	276 (15.36)	56 (16.67)	220 (15.06)	0.54 (χ^2^)	0.461
No, n (%)	1521 (84.64)	280 (83.33)	1241 (84.94)		
**Joint target control in 2011**					
Yes, n (%)	275 (15.30)	48 (14.29)	227 (15.54)	0.33 (χ^2^)	0.566
No, n (%)	1522 (84.70)	288 (85.71)	1234 (84.46)		
**Joint target control in 2013**					
Yes, n (%)	490 (27.27)	79 (23.51)	411 (28.13)	2.94 (χ^2^)	0.086
No, n (%)	1307 (72.73)	257 (76.49)	1050 (71.87)		
**Joint target control in 2014**					
Yes, n (%)	602 (33.50)	101 (30.06)	501 (34.29)	2.20 (χ^2^)	0.138
No, n (%)	1195 (66.50)	235 (69.94)	960 (65.71)		
**Joint target control in 2015**					
Yes, n (%)	569 (31.66)	93 (27.68)	476 (32.58)	3.03 (χ^2^)	0.082
No, n (%)	1228 (68.34)	243 (72.32)	985 (67.42)		
**Joint target control in 2016**					
Yes, n (%)	516 (28.71)	68 (20.24)	448 (30.66)	14.51 (χ^2^)	< 0.001
No, n (%)	1281 (71.29)	268 (79.76)	1013 (69.34)		
**Joint target control in 2017**					
Yes, n (%)	454 (25.26)	64 (19.05)	390 (26.69)	8.46 (χ^2^)	0.004
No, n (%)	1343 (74.74)	272 (80.95)	1071 (73.31)		
**Joint target control in 2018**					
Yes, n (%)	369 (20.53)	56 (16.67)	313 (21.42)	3.79 (χ^2^)	0.052
No, n (%)	1428 (79.47)	280 (83.33)	1148 (78.58)

For glucose metabolism parameters and a number of the hemodynamic values (lipid and serum creatinine concentrations), after 10-year follow-up, there were no significant differences in these variables between the two groups (Table 2).

### Effect of acarbose therapy on the incidence of CVD events

**Figure 3:**
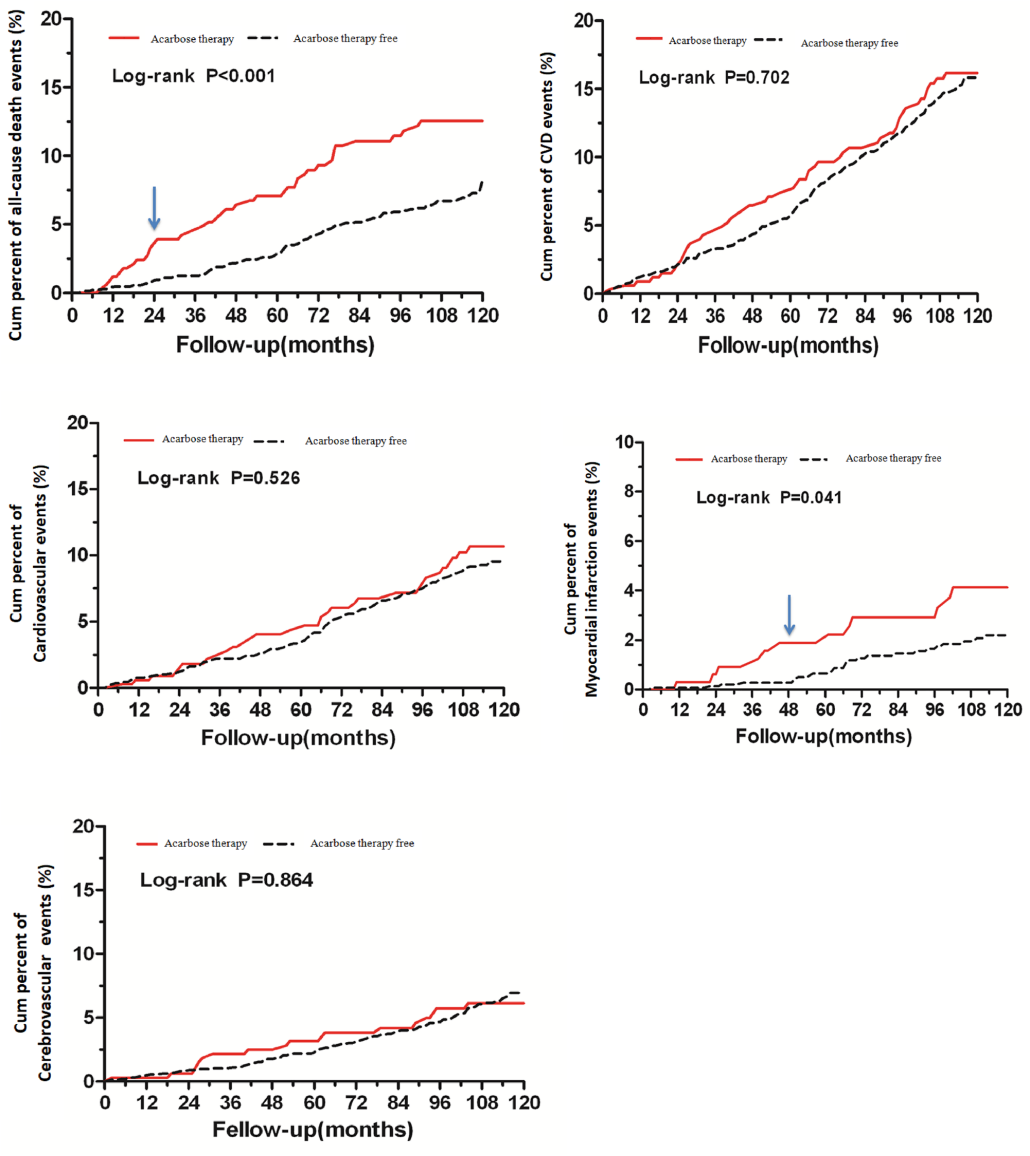
Cumulative incidences of all-cause end-point events during the follow-up between the two groups. The incidences of myocardial infarction (from the 4th year of follow-up) and all-cause death (from the 2nd year of follow-up) in patients who received acarbose therapy were significantly lower than that of acarbose therapy free respectively.

During the 10-year follow-up, a total of 446 endpoint events occurred, including all-cause death, cardiovascular events, cerebrovascular events and tumor. 199 patients (13.62%) who had received acarbose treatment developed CVD as compared to 49 patients (14.58%) who never took acarbose therapy. Furthermore, 81 patients (5.54%) with acarbose treatment developed cerebrovascular disease as compared to 18 patients (5.36%) without acarbose therapy. Compared with acarbose therapy free, acarbose users had a lower incidence of myocardial infarction events (1.78% vs. 3.57%, P < 0.05) and all-cause death (6.16% vs. 11.61%, P < 0.01) (Table 4). The incidences of myocardial infarction (from the 4th year of follow-up) and all-cause death (from the 2nd year of follow-up) in patients who received acarbose therapy were significantly lower than that of acarbose therapy free respectively (Fig. 3).

**Table 4:** The incidence of all-cause end-point events between the two groups.

End-point events	Acarbose therapy free	Acarbose therapy	Statistical quantity	P
	(n = 336)	(n = 1461)		
**All-cause death**				
No, n (%)	297 (88.39)	1371 (93.84)	12.16 (χ^2^)	< 0.001
Yes, n (%)	39 (11.61)	90 (6.16)		
**Cardiac and cerebral vascular disease**				
No, n (%)	287 (85.42)	1262 (86.38)	0.21 (χ^2^)	0.645
Yes, n (%)	49 (14.58)	199 (13.62)		
**Cardiovascular disease**				
No, n (%)	305 (90.77)	1343 (91.92)	0.47 (χ^2^)	0.491
Yes, n (%)	31 (9.23)	118 (8.08)		
**Myocardial infarction**				
No, n (%)	324 (96.43)	1435 (98.22)	4.24 (χ^2^)	0.040
Yes, n (%)	12 (3.57)	26 (1.78)		
**Cerebrovascular disease**				
No, n (%)	318 (94.64)	1380 (94.46)	0.02 (χ^2^)	0.892
Yes, n (%)	18 (5.36)	81 (5.54)		
**Malignant tumor**				
No, n (%)	319 (94.94)	1409 (96.44)	1.67 (χ^2^)	0.197
Yes, n (%)	17 (5.06)	52 (3.56)

In comparison to acarbose therapy free, the cumulative cardiovascular event rate was significantly lower in patients with acarbose therapy (*P* < 0.001) (Fig. 3). No first cardiovascular events were diagnosed only by ECG, as all had previously been diagnosed clinically.

There were no significant differences in the survival ratio of patients with previous CVD between acarbose therapy group and acarbose therapy free group (Table 5).

**Table 5:** Survival ratio of patients with previous CVD in different acarbose treatment groups.

	Acarbose therapy free		Acarbose therapy		Statistical quantity	P
	Survivors	Deaths	Survivors	Deaths		
**Cardiac and cerebral vascular disease**						
No, n (%)	211 (90.56)	22 (9.44)	985 (95.82)	43 (4.18)	10.75 (χ^2^)	0.001
Yes, n (%)	86 (83.50)	17 (16.50)	383 (88.45)	50 (11.55)	1.87 (χ^2^)	0.172
**Cerebral vascular disease**						
No, n (%)	266 (89.56)	31 (10.44)	1203 (94.87)	65 (5.13)	11.79 (χ^2^)	0.001
Yes, n (%)	31 (79.49)	8 (20.51)	165 (85.49)	28 (14.51)	0.89 (χ^2^)	0.345
**Coronary heart disease**						
No, n (%)	236 (90.08)	26 (9.92)	1092 (95.12)	56 (4.88)	9.91 (χ^2^)	0.002
Yes, n (%)	61 (82.43)	13 (17.57)	276 (88.18)	37 (11.82)	1.76 (χ^2^)	0.185

Factors analysis was performed to explore the variables contributing to the incidence of 10-year all-cause endpoint events. After adjusting for age, gender, smoking, educational attainment, application of hypolipidemic drugs use (including Statins) and concomitant use of other antidiabetic regimens, by using those without exposure to acarbose as reference value, during the 10-year follow-up, the crude (95% CI) hazard ratio (HR) of acarbose users in all-cause death was 0.53 (95% CI 0.37–0.78, P = 0.001) (Table 6). Further analysis indicated that adjusted HR of acarbose users in myocardial infarction was 0.49 (95% CI 0.25–0.97, P < 0.05) (Table 6).

**Table 6:** Cox regression of the effect of acarbose therapy on various endpoint events in BCDS study.

	b	std	Wald	P	HR	95% CI for HR	
						Lower	Upper
All-cause death	− 0.63	0.19	10.34	0.001	0.53	0.37	0.78
Cardiac and cerebral vascular disease	− 0.11	0.16	0.50	0.477	0.89	0.65	1.22
Cardiovascular disease	− 0.17	0.20	0.67	0.413	0.85	0.57	1.26
Myocardial infarction	− 0.72	0.35	4.14	0.042	0.49	0.25	0.97
Cerebrovascular disease	− 0.03	0.26	0.01	0.909	0.97	0.58	1.62
Malignant tumor	− 0.37	0.28	1.69	0.193	0.69	0.40	1.20

## Discussion

Most evidence-based studies implied that compared with fasting blood glucose level, postprandial blood glucose level might be related to chronic complications in T2DM more closely. Previous studies have indicated that postprandial hyperglycemia seems to play an important role in cardiovascular complications development in T2DM^13,14^. Especially in Asian diabetic subjects, postprandial hyperglycemia contributes more prominently to the level of HbA1c^15^.

Although treatment strategies in T2DM are increasingly updated, but the optimum strategy for glycemic control remains controversial. Chinese population is dominated by the traditional high-carbohydrate dietary pattern. Acarbose, an oral antidiabetic agent, could competitively inhibit α-glucosidases absorption in small intestine. Since carbohydrate absorption and digestion occur throughout the small intestine, acarbose is used to delay sugar absorption to reduce postprandial blood glucose^7^. A lot of experience in the application of acarbose has been obtained in clinical research, which can be used alone or in combination with other oral hypoglycemic drugs and insulin, especially in the Chinese T2DM guidelines^16,17^.

Although recent study once focused on the potential benefits of acarbose treatment of cardiovascular disease (CVD) in patients with T2DM by using the Taiwanese National Health Insurance Research Database (NHIRD), there were no retrieve clinical data like lipids and glycemic, blood pressure control from this claim dataset^18^.

Since little information exists about the potential cardiovascular effect of α-glucosidase inhibitor use in Asian populations; we aimed to ascertain the effectiveness of the acarbose on the risk of cardiovascular events in T2DM, extensively adopted in China. This is the only 10-year study in oral antidiabetic drugs that reduces myocardial infarction events.

The main findings from the present study indicated that acarbose therapy, either as monotherapy or in combination with other antidiabetic regimens in T2DM, provided an important impact on the subsequent development of CVD. Specifically, during the 10 years follow-up, the relative risk of myocardial infarction reduction was similar at 50% in patients with acarbose therapy. Acarbose therapy has the potential to prevent deaths from complications related to diabetes as cardiovascular and cerebrovascular disease account for 52% of all mortality. Even after adjusting for major risk factors, the reduction in the risk of cardiovascular events associated with acarbose treatment was still statistically significant. From the 2nd year of follow-up, the incidences of all-cause death in patients who received acarbose therapy were significantly lower than that of acarbose therapy free; while from the 4th year of follow-up, the patients received acarbose therapy had lower incidences of myocardial infarction than that of non-users (Fig. 3).

Analysis of the clinical characteristics, such as age, gender, the frequency of smoking, comorbidities and combination treatment showed similar patterns between the two groups at the baseline. Indeed, compared with patients with acarbose therapy free, the acarbose users had higher educational attainment and more use of insulin therapy. To our knowledge, previous study explored acarbose were associated with significant insulin-sparing effects in both the fasting state and after standard meal test^19^.

The changes in postprandial glucose with acarbose treatment were in agreement with previous studies^20^. In the present study, at the end of follow-up, compared with the baseline, the mean levels of 2-h postprandial blood glucose and HbA1c decreased, while the fasting blood glucose levels increase. These results showed a light that the improvement of HbA1c was mainly due to the control of postprandial blood glucose in our study. Therefore, acarbose might be involved in preventing the onset and progression of diabetic chronic complications by reducing postprandial blood glucose. This needs to be further confirmed by prospective studies.

In addition, the present study showed that many metabolic indicators (including blood pressure, and LDL-cholesterols) were significantly improved in both acarbose therapy and acarbose therapy free after 10 years multi-factorial invention when compared with baseline. These findings are in agreement with results from the previous studies^21^. With respect to the reduction of SBP by acarbose, although many previous studies have provided evidence for an effect^22^, it is still unclear.

A meta-analysis to assess if treatment with the α-glucosidase inhibitor acarbose can reduce cardiovascular events in T2DM showed that body weight improved significantly during acarbose treatment^9^. Acarbose’s effects on body weight reduction in this study are in line with those of previous studies in Chinese^23^ and other populations^24^. Overweight/obesity is recognized as one of the most common risk factors for CVD. Neck circumference was shown in many studies to be an indicator for evaluating overweight/obesity^25^, and was associated with the occurrence of cardiovascular events in type 2 diabetes in Chinese communities, and may increase the risk of cardiovascular events by about 2.3-fold^26^. In the present study, the neck circumference was significant higher in patients with acarbose therapy free than that of acarbose therapy, while after 10 year-follow-up, there were no significant difference between the two groups. We speculated that individuals with overweight/obesity might be most likely to benefit from reduction of weight as they are particularly at risk from the myocardial infarction risk of T2DM. However, the underlying mechanisms of weight reduction by acarbose and its clinical relevance need to be examined further.

There is growing evidence that joint target control plays an important role in the development of diabetes complications^27,28^. What's important is this article differs from other research is the 10 years’ observation, which explained the relationship between acarbose therapy and myocardial infarction. At baseline, compared with the acarbose therapy free in T2DM, there was no significant difference in achieving the joint target control in patients with acarbose therapy. However, from the beginning of 8th year follow-up (2016), the joint target control rate in patients with acarbose therapy (30.66%) was significantly higher than that of acarbose therapy free (20.24%). This trend was maintained in the following years (Fig. 2). The benefits on CVD began to emerge in patients with acarbose therapy.

When we explored the relationship between acarbose therapy and myocardial infarction and all-cause death at the same time, actually, our study indicated that the use of acarbose could rapidly improve joint target control. On the other hand, diabetic subjects who used acarbose showed benefits of protection, both in myocardial infarction and all-cause death. Therefore, we suggested that improving joint target control might be one of the main reasons of myocardial infarction and all-cause death reduction. The exact mechanisms definitely require further investigations.

In the present study, Cox proportional hazards analysis revealed that metabolic score (MS) of blood pressure, HbA1c and acarbose therapy were independent predictive factors for CVD events in T2DM. Acarbose therapy was negatively correlated with CVD disease. Intervention with acarbose can prevent myocardial infarction and cardiovascular disease in T2DM while most of them are already on intensive concomitant.

## Conclusions

To our knowledge, this is the first study to evaluate the effect of acarbose therapy on the risk of cardiovascular events in T2DM during 10-year multifactorial interventions. Otherwise, this study was a well-documented clinical trial, which increase the reliability of our findings.

Our study suggests that treatment with acarbose in T2DM revealed a relatively pattern of benefits on developing CVD. After the 10 years’ multifactorial interventions, in patients with acarbose therapy, the relative risk of myocardial infarction reduction was similar at 50% and all-cause death reduction was about 52%. The effects of acarbose on improving joint target control might be one of the main reasons of CVD events reduction. This study was only studied in Chinese population and not in non-Chinese population. So the data was only as a suggestion to the Chinese population.

## Limitations

Because the population in this study requires a follow-up of 10 years, unfortunately, patients with less than 10 years of follow-up were not included in this study population. At the same time, patients without medication information were also excluded in the statistics of this article. Due to afore reasons, the samples of patients in this study were limited. Since the food of Chinese is mainly carbohydrates, Chinese people are more suitable for acarbose treatment. Acarbose therapy is safe and the incidence of hypoglycemia is low. This study is not a double-blind controlled study, based on the actual data of patients, the number of patients receiving and not receiving acarbose might be significantly different.

## Data Availability

The datasets during and/or analyzed during the current study are available from the corresponding author on reasonable request.
