# Author Correction: The effects of acarbose therapy on reductions of myocardial infarction and all-cause death in T2DM during 10-year multifactorial interventions (The Beijing Community Diabetes Study 24)

**DOI:** 10.1038/s41598-021-90670-0

**Published:** 2021-05-20

**Authors:** Xue-Lian Zhang, Shen-Yuan Yuan, Gang Wan, Ming-Xia Yuan, Guang-Ran Yang, Han-Jing Fu, Liang-Xiang Zhu, Jian-Dong Zhang, Yu-Ling Li, Da-yong Gao, Xue-Li Cui, Zi-ming Wang, Rong-Rong Xie, Ying-jun Chen

**Affiliations:** 1 Department of Endocrinology, Beijing Tongren Hospital, Capital Medical University, 1 Dong Jiao Min Xiang, Beijing, 100730 China; 2 Medical Records and Statistics Department, Beijing Ditan Hospital, Capital Medical University, Beijing, China; 3 Department of Endocrinology, Beijing Friendship Hospital, Capital Medical University, Beijing, China; 4 Jinsong Community Health Service Center, Beijing, China; 5 Xinjiekou Community Health Service Center, Beijing, China; 6 Aerospace Central Hospital, Beijing, China; 7 Sanlitun Community Health Service Center, Beijing, China; 8 Jiangtai Community Health Service Center, Beijing, China; 9 Majiapu Community Health Service Center, Beijing, China

Correction to: *Scientific Reports* 10.1038/s41598-021-84015-0, published online 01 March 2021

This Article contains an error in Figure 3 labelling of the endpoint events in Non-Acarbose users (red solid line) and Acarbose users (grey dashed line).

The correct Figure 3 appears below as Figure 1.

**Figure 1:**
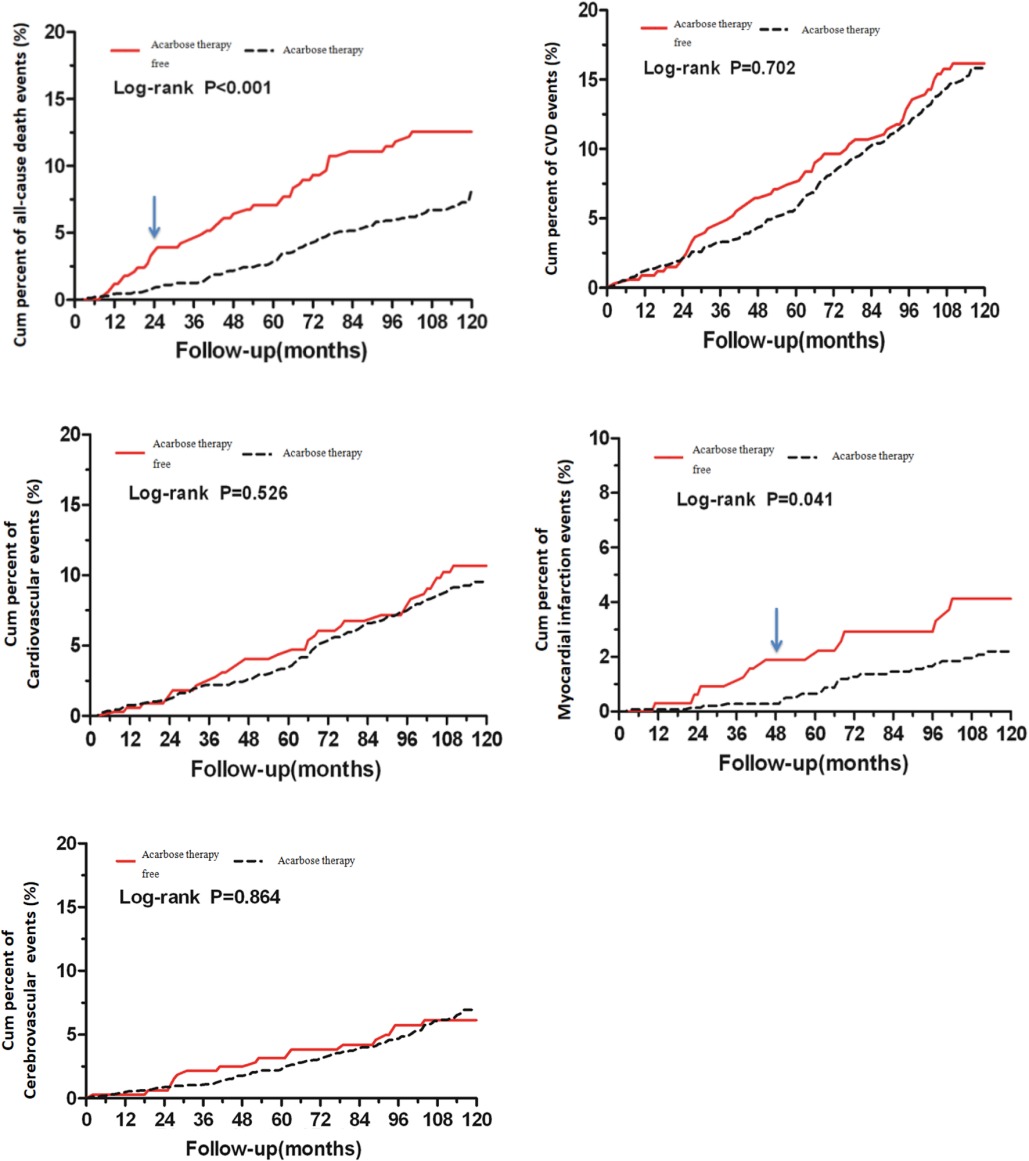
A correct version of the original Figure 3.

